# A placental DNA methylation score associated with early-onset preeclampsia and maternal vascular malperfusion pathology

**DOI:** 10.3389/freae.2026.1749513

**Published:** 2026-06-05

**Authors:** Hannah J. Illing, Alexa A. Freedman, Lauren Keenan-Devlin, Gregory E. Miller, Ann E. Borders, Linda M. Ernst, Wendy P. Robinson

**Affiliations:** 1 Department of Medical Genetics, University of British Columbia, Vancouver, BC, Canada; 2 British Columbia Children’s Hospital Research Institute (BCCHR), Vancouver, BC, Canada; 3 Department of Psychology, Northwestern University, Evanston, IL, United States; 4 Endeavor Health, Evanston, IL, United States; 5 University of Chicago Pritzker School of Medicine, Evanston, IL, United States

**Keywords:** DNA methylation, maternal vascular malperfusion, pathology, placenta, preeclampsia

## Abstract

**Introduction:**

Early-onset preeclampsia (EOPE) is a severe maternal hypertensive disorder of pregnancy, where placental dysfunction is a component of the pathophysiology. Previously, we developed the eoPRED tool, which is a DNA methylation (DNAme)-based method that estimates the likelihood that a placenta came from a pregnancy complicated by EOPE. We hypothesize that this score may reflect maternal vascular malperfusion (MVM), which is the most common placental pathology observed in early-onset preeclampsia (EOPE), but is also associated with preterm birth and fetal growth restriction. To test this we evaluated whether eoPRED score is associated with the severity of MVM pathology in placentas from both preeclamptic and non-preeclamptic pregnancies.

**Methods:**

We utilized 493 placentas with both Infinium EPICv1.0 DNA methylation data and histopathologic characterization by a placental pathologist. We evaluated associations between eoPRED score and four major classes of placental pathology: maternal vascular malperfusion (MVM), fetal vascular malperfusion (FVM), chronic inflammation (CI), and acute inflammation (AI).

**Results:**

eoPRED score was higher in placentas from pregnancies with EOPE (n = 7) than controls without preeclampsia (n = 402, p < 0.001). Independent of EOPE, eoPRED correlated with the grade of MVM (R = 0.34, p < 0.01), but was not associated with any other pathology class.

**Discussion:**

eoPRED score can not only be used to identify cases of likely EOPE but is also associated with placental maternal vascular dysfunction in the absence of preeclampsia. This score may thus be useful in placental DNAme datasets lacking pathology information to identify associations between exposures/and or birth outcomes with placental dysfunction.

## 1 Introduction

Preeclampsia is a serious hypertensive pregnancy condition that is a leading cause of maternal and fetal morbidity and mortality, and estimated to occur in 2–8% of pregnancies globally (Abalos et al., 2013; Clark et al., 2008; Dzakpasu et al., 2024). The contributing factors to preeclampsia are multifactorial (maternal morbidities, genetics, etc.) although poor or reduced placental perfusion and its downstream consequences are thought to underly the most severe forms of preeclampsia (Kovo et al., 2012; Staff, 2019). Preeclampsia is often clinically separated into early-onset (EOPE) and late-onset (LOPE), depending on whether the onset occurred before or after 34-week gestation (Raymond and Peterson, 2011). Although this distinction is not clear-cut, in EOPE maternal symptoms are generally more severe, neonate outcomes are worse, and placental pathology is typically present (Freedman et al., 2023; Freedman et al., 2021). In contrast, LOPE does not have as clear associations with placental pathology (Freedman et al., 2023; Roberts et al., 2021).

Numerous groups have studied molecular associations in the placenta using transcriptomic, genomic, and epigenomic data (Freedman et al., 2023; Ren et al., 2021; Leavey et al., 2018; Wang et al., 2020; Cirkovic et al., 2020) in an attempt to explore deeper into the pathophysiology underlying preeclampsia. There is a tendency to lower methylation in placentas from preeclamptic relative to non-preeclamptic pregnancies, and there are more changes in the placental methylome associated with EOPE than LOPE (13–15). However, there have been few studies linking such molecular changes directly to placental histopathology findings. Maternal vascular malperfusion (MVM) is the most common placental pathology observed in placentas from pregnancies with EOPE, being present in 70–95% of cases (Freedman et al., 2023; Zur et al., 2024; Ogge et al., 2011). MVM represents a broad category of placental pathology defined by damage to utero-placental vasculature resulting in impaired maternal blood flow to the placenta, and is characterized by lesions to the chorionic villi that can include placental infarction, retroplacental hemorrhage, accelerated villous maturation, and distal villous hypoplasia (Khong et al., 2016). While MVM is associated with preeclampsia, it is also prevalent in cases of fetal growth restriction and preterm birth (Freedman et al., 2023; Agrawal et al., 2022), and is even relatively common in placentas from uncomplicated pregnancies (12–36%) (Falco et al., 2017; Romero et al., 2018). If the molecular signatures associated with EOPE are also linked to MVM, it could provide insight into the mechanisms underlying EOPE as well as other MVM-related pregnancy outcomes.

DNA methylation (DNAme) is one molecular tool that has been widely used to study EOPE in the placenta post-delivery (Cirkovic et al., 2020; Wilson et al., 2018; Lim et al., 2020; Chu et al., 2014; Wang et al., 2019; Herzog et al., 2017). Recently, Boyano et al. (Fernández-Boyano et al., 2023) developed *eoPRED*, a machine learning-based classification tool that utilizes DNAme at 45 CpGs to score a placental DNA methylome from zero to one, corresponding to the probability of the placenta coming from pregnancy with EOPE. eoPRED was initially developed with the idea to use DNAme to improve identification of an EOPE or “placental preeclampsia” phenotype. However, the eoPRED tool was trained using the clinical diagnosis of EOPE and has not yet been evaluated in relationship to placental histopathology findings. We hypothesized that the eoPRED score may reflect molecular changes associated with MVM and would therefore correlate with the severity of MVM pathology. If so, this could offer an approach to quantify an aspect of EOPE associated placental pathology, independent of the presence of a clinical diagnosis of EOPE. Thus, our objective was to explore associations between eoPRED score and placental histopathology.

## 2 Methods

### 2.1 Cohort and clinical variables

A summary flowchart of our methods is in Supplementary Figure S1. We utilized Infinium MethylationEPIC BeadChipv1.0 array (Illumina, USA) data from 493 placentas in the Stress Pregnancy and Health Cohort (SPAH) (GSE307289, Chicago, US) (Keenan‐Devlin et al., 2025; Beraldo et al., 2025; Illing et al., 2026). The dataset has been described in detail in previous papers (Keenan‐Devlin et al., 2025; Beraldo et al., 2025; Illing et al., 2026), and included placentas from pregnant individuals >18 years of age with singleton pregnancies. Individuals were excluded from the study if there were fetal congenital anomalies or known chromosomal abnormalities in the pregnancy.

This data was based on DNA pooled from three ∼0.4 cm^3^ chorionic villi biopsies, each taken from beneath the fetal-facing surface from separate cotelydons, avoiding areas of obvious pathology. DNA was extracted from the three sites and pooled for DNAme analysis. Gestational age was estimated by last menstrual period and confirmed with ultrasound. Diagnoses of preeclampsia and gestational hypertension were defined according to the American College of Obstetricians and Gynecologists and abstracted from electronic medical records (Author Anonymous, 2020). Gestational hypertension was defined as systolic blood pressure ≥140 mmHg and/or diastolic blood pressure ≥90 mmHg on two occasions ≥4 h apart after 20 weeks gestation. Preeclampsia was defined as gestational hypertension accompanied by proteinuria or a severe feature (e.g., thrombocytopenia, renal insufficiency). Samples were further separated into EOPE (<34 weeks) and late-onset preeclampsia (LOPE) (≥34 weeks) based on gestational age at birth, since gestational age at diagnosis was unavailable. A limitation to this approach is that a subset of EOPE cases could be classified as LOPE (i.e., if diagnosed with preeclampsia before 34 weeks but delivering after 34 weeks), however all cases classified as EOPE will be true EOPE. Non-preeclamptic placentas (including those with gestational hypertension) were separated into non-preeclamptic preterm birth (PTB, <37 weeks) and non-preeclamptic term birth (TB, ≥37 weeks). There were 2 samples without preeclampsia diagnosis information which we excluded from all analyses. There were 8 other samples without pathology classification, and these samples were excluded only from analyses where that information was needed.

### 2.2 Placental pathology assessment

Placentas underwent detailed histopathological review by an experienced perinatal pathologist (LME). Using up-to-date definitions placental lesions were categorized into the four major categories: acute inflammation (AI), chronic inflammation (CI), fetal vascular malperfusion (FVM), or MVM (Freedman et al., 2021; Khong et al., 2016). AI is defined by neutrophilic infiltration of the amnion, chorion, chorionic plate, umbilical cord or chorionic vessels. CI included the abnormal presence of lymphocytes, histiocytes or plasma cells in any compartment of the placenta. FVM included thrombosis of at least one fetal vessel and/or avascular villi. MVM was defined by the presence of decidual vascular lesions, retroplacental hematoma, and villous lesions such as infarction, accelerated villous maturation and distal villous hypoplasia (see supplementary methods). MVM was scored based on the number of lesions, with more severe lesions receiving two points. Scores of 2-3 were considered low-grade, and scores over 4 were considered high-grade (Freedman et al., 2021).

### 2.3 DNAme processing and DNAme estimated variables

Beta-values were normalized with the *dasen* and *noob* normalization (Pidsley et al., 2013; Triche et al., 2013). We followed the processing pipeline detailed in Khan et al., 2023 (Khan et al., 2023), which included detailed sample and probe quality checks and the removal of the following CpGs: genotyping (n = 59), sex chromosome (n = 16,928), polymorphic and cross-hybridizing (n = 105,454) (Zhou et al., 2017), those with known manufacturing issues (n = 1,369) (Illumina, 2020), and detection P > 0.01 or beadcount <3 in ≥5% of samples (n = 12,737).

Using the *PlaNET* R package (Yuan and Robinson, 2024) we estimated eoPRED score (Fernández-Boyano et al., 2023) and cell-type estimates (Yuan et al., 2021). eoPRED was designed using a machine learning approach as a binary classifier (EOPE/PTB) with eoPRED score (range 0–1) corresponding to a sample’s probability of coming from an pregnancy with EOPE (Fernández-Boyano et al., 2023). It was trained on 42 cases of EOPE and validated on an additional 38 EOPE cases, in comparison to normotensive preterm births of similar gestational age. The 45 CpGs that contribute to eoPRED score were selected by sparse partial least squares discriminant analysis. Some of these 45 CpGs are associated with genes for which expression is known to be altered in preeclampsia (*PAPPA2, PPARG, FLNB*), but many of the sites are intragenic and of no known function, as they were chosen agnostic to function. As the score was developed comparing preeclampsia cases with gestational age matched controls it is independent of gestational age. The PlaNET robust partial correlations method uses cell-type distinct markers to estimate the proportions of the six major placental cell-types: syncytiotrophoblast, cytotrophoblast, stromal, endothelial, Hofbauer, and nucleated red blood cells (nRBC).

### 2.4 Statistics

Pearson correlation was used to test for linear associations between variables. Kruskal–Wallis (non-parametric) test followed by pairwise Dunn’s test with false discovery rate (FDR) adjustment was used to test for differences in group means. To test eoPRED associations with cell composition, linear regressions were run with eoPRED score as the dependent variable and gestational age as a covariate with each of the cell-type estimates, as placental cell composition changes with gestational age.

## 3 Results

### 3.1 eoPRED score is associated with EOPE

Before evaluating eoPRED score in the context of pathology, we assessed the ability of eoPRED score to identify placentas from pregnancies affected by EOPE in the SPAH dataset (n = 7). The mean eoPRED score for placentas from EOPE affected pregnancies (x̄ = 0.69) was significantly higher than the mean eoPRED score of the PTB (n = 40, x̄ = 0.39) and TB (n = 402, x̄ = 0.25) groups (Figure 1A). Placentas from LOPE (n = 42) affected pregnancies also had a higher mean eoPRED score (x̄ = 0.46) than TB cases (p < 0.001). In the development of the eoPRED score, a cut-off of >0.55 was suggested to be used to classify samples as EOPE or PTB (Fernández-Boyano et al., 2023). In the SPAH data 5/7 (71.4%) cases of EOPE and 9/42 (21.4%) of LOPE had scores >0.55, while only 19/442 (4.3%) non-preeclamptic cases scored this high. Furthermore, 3 of the 7 EOPE cases displayed higher scores than any of the remaining nearly 500 placentas evaluated, confirming a strong association of this score with EOPE.

**FIGURE 1:**
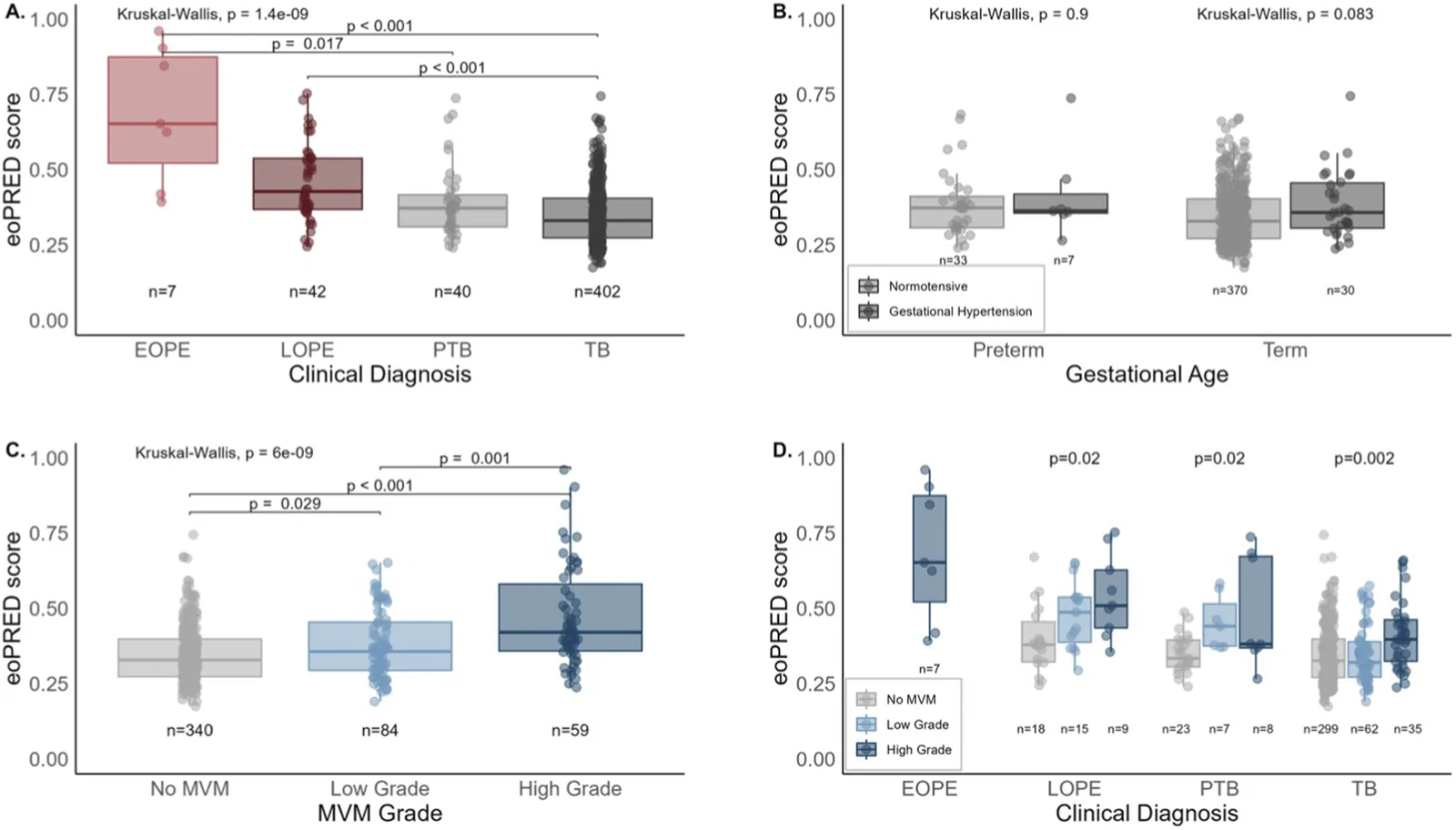
eoPRED score associates with EOPE and MVM pathology. **(A)** eoPRED score plotted by clinical diagnosis of early-onset preeclampsia (EOPE, n = 7), late-onset preeclampsia (LOPE, n = 42), non-preeclamptic preterm births (PTB, n = 40), and non-preeclamptic term births (TB, n = 402); Kruskal–Wallis test followed by Dunn Test with FDR adjustment, only significant differences shown. **(B)** eoPRED score was not different between placentas from pregnancies with and without gestational hypertension in the PTB or TB groups (Kruskal–Wallis test). **(C)** Grade of MVM is associated with higher eoPRED scores; Kruskal–Wallis test followed by Dunn Test with FDR adjustment, only significant differences shown. **(D)** Grade of MVM is associated with higher eoPRED scores within the diagnosis groups (Kruskal–Wallis test).

A high eoPRED score could be a consequence of the maternal disease in turn affecting placental function and so we next we asked whether eoPRED score was associated with just maternal hypertension. To assess this, we compared placentas from pregnancies with gestational hypertension, but no preeclampsia, to gestational-age matched non-hypertensive controls. While there were slightly higher eoPRED scores in the gestational hypertension groups, this difference was not significant (p > 0.05, Figure 1B).

### 3.2 eoPRED score is associated with MVM pathology

A high eoPRED score may also reflect an underlying placental pathology that increases the risk for EOPE, and so we next assessed whether this score was linked to placental pathology. The composition of pathologies in the SPAH dataset is discussed in detail in Illing et al. (2026) (28) and includes placentas presenting with MVM (n = 143, 29.5%), FVM (n = 162, 33.4%), AI (n = 267, 55.1%), and CI (n = 266 each, 54.9%), no pathology (n = 44). Placentas could have multiple lesion types. High-grade MVM was present in all 7 cases of EOPE, but MVM was also present in 24/42 (57.1%) cases of LOPE, 15/38 non-preeclamptic preterm (39.5%) and 97/396 term (24.5%) births. Thus, the majority (79%, 113/143) of MVM cases were not associated with a clinical diagnosis of EOPE or LOPE.

The mean eoPRED score was higher in all placentas with MVM (n = 143, x̄ = 0.42) compared to placentas without MVM (n = 340 x̄ = 0.35, p < 0.001, Wilcoxon test). We then excluded samples with EOPE and the mean eoPRED score remained higher in placentas with MVM (n = 136, x̄ = 0.40, p < 0.001). Furthermore, eoPRED score was notably higher in placentas with high-grade MVM compared to placentas with low grade or no MVM (Figure 1C) and this held within each of the diagnosis groups (Figure 1D).

We then tested whether eoPRED score had a stronger association with a particular histopathologic characteristic of MVM. eoPRED was positively correlated with all histopathologic lesions that are characteristic of MVM other than perivillous fibrin deposition which was not correlated. eoPRED had the strongest correlation with distal villous hyperplasia, infarcts, and syncytial knots (Supplementary Figure S2). eoPRED score appears to be specific to MVM pathology, as it was not associated with the presence/absence of AI, CI, or FVM pathologies (Supplementary Figure S2).

### 3.3 All CpGs composing the eoPRED score are associated with MVM pathology

We regressed MVM (presence/absence) on each of the 45 CpGs that contribute to the eoPRED score to assess whether MVM pathology was strongly associated with all or only a subset of the CpGs in the tool. Multiple test correction with FDR adjustment was performed and FDR adjusted p-values were analyzed. All 45 CpGs were associated with MVM (FDR<0.05) and had lower DNAme in samples with MVM than those without; however, the magnitudes of the regression coefficients were quite small for most CpGs (|Δβ|<0.05, Supplementary Table S1). The two CpGs with effect size |Δβ|>0.05 included cg23730027 (chr3: 58009452) located in the *FLNB* promoter and cg26625897 (chr17: 41522426) located in an enhancer just upstream from the *KRT15* transcription start site.

### 3.4 eoPRED score associates with altered cell composition

As molecular changes associated with EOPE can reflect in part differences in placental cell composition (Redline and Patterson, 1995; Campbell et al., 2023; Inkster et al., 2025), we next investigated whether eoPRED score associated with altered cell composition. Although the CpGs used in the eoPRED tool were reported to be independent of cell-type estimates, multiple datasets were combined in the initial study, each with varying cell compositions which may have reduced sensitivity to detect such effects. Based on linear regression, eoPRED score was positively associated with syncytiotrophoblast proportion and nRBC estimates and negatively associated with cytotrophoblast, endothelial, stromal, and Hofbauer cell estimates (Table 1). The syncytiotrophoblast had a smaller regression coefficient (0.0025) than other cell estimates; however, syncytiotrophoblast cells make up the largest proportion of samples in this dataset (x̄ = 0.82, SD = 0.09, Table 1) and it is the most variable cell proportion.

**TABLE 1:** eoPRED score is associated with PlaNET estimated placental cell proportions.

Cell type	Δ eoPRED score for 1% change in cell estimate	95% confidence interval	p-value	Proportion of sample (mean, (SD))
Syncytiotrophoblast	0.0025	0.002–0.003	<0.001	0.82 (0.09)
Cytotrophoblast	−0.0023	−0.004–−0.0005	0.01	0.06 (0.06)
Endothelial	−0.0115	−0.015–−0.008	<0.001	0.06 (0.02)
Stromal	−0.0114	−0.015–−0.0082	<0.001	0.03 (0.03)
nRBC	0.0210	0.013–0.029	<0.001	0.02 (0.01)
Hofbauer	−0.0233	−0.034–−0.013	<0.001	0.009 (0.009)

## 4 Discussion

In this study we demonstrated that eoPRED score, a molecular tool developed to classify placentas as EOPE, was not only associated with the clinical diagnosis of EOPE, but also was associated with placental maternal vascular dysfunction in the absence of a diagnosis of preeclampsia. While predicting presence/or absence of EOPE may have utility, eoPRED score has additional value as it can provide a quantitative measure that may reflect underlying MVM pathology.

In this study, eoPRED score was higher in placentas from EOPE associated pregnancies, but not those with gestational hypertension, supporting the hypothesis that gestational hypertension is often due to maternal rather than placental factors (Staff, 2019) and is not the cause of DNAme changes in the placenta associated with this score. While EOPE cases did have higher mean eoPRED scores than LOPE cases, 21 of the LOPE cases had eoPRED scores >0.55, which likely represents that a subset of these cases share similar placental pathology to EOPE. Using eoPRED score as a continuous variable rather than discrete EOPE/LOPE categories may better reflect the degree to which placental pathology contributes to disease presentation.

Independent of preeclampsia diagnosis, eoPRED score was associated with the presence and grade of MVM pathology, but no other placental pathology findings. While the correlation was moderate (R ≈ 0.34), we cannot distinguish whether a molecular approach or clinical assessment of placental lesions, which has some subjectivity, more accurately reflects underlying placental function. This aligns with previous studies that examined transcriptomic data of placentas from pregnancies with preeclampsia (Benton et al., 2018; Leavey et al., 2016). Placentas clustered based on their transcriptomic profiles into a “canonical preeclampsia” cluster which was strongly associated with the presence MVM. Within this cluster, the grade of MVM pathology correlated with a more distinct transcriptomic profile from non-preeclamptic cases. Out study similarly identified the correlation between the severity of MVM pathology and the extent of molecular changes and adds that eoPRED score can be used as a quantitative measure of that severity.

MVM was associated with all probes in the eoPRED predictor, however the two probes most strongly associated with MVM were present in or adjacent to the genes *FLNB* and *KRT15*, both of which encode components of the cytoskeleton. DNAme or gene expression changes at *FLNB* and *KRT15* have been reproducibly associated with EOPE, and they may be involved in placental angiogenesis (Wilson et al., 2018; Liu et al., 2024; Huang et al., 2022; Blair et al., 2013).

eoPRED score was also associated with higher syncytiotrophoblast cells and nRBC and lower estimated cytotrophoblast, stromal, endothelial, and Hofbauer cell estimates. This aligns with deconvoluted transcriptomic (Campbell et al., 2023) and DNAme (Inkster et al., 2025) data which found EOPE to be associated with lower mesenchymal (stromal and endothelial) and Hofbauer cell estimates. The reduction in villous core cells detected by DNAme reflects that MVM is characterized by increased syncytial knots and smaller and less vascular villi (Khong et al., 2016). These were also the features of MVM pathology that eoPRED score was most strongly associated with.

Strengths of this study included using a single large dataset evaluated by one pathologist to avoid interobserver variability or batch effects and a standardized placental sampling protocol. A limitation is that placentas were biopsied avoiding areas of obvious lesions, thus we may have underestimated the difference in eoPRED score associated with MVM. Although MVM grade correlated with higher eoPRED score there was considerable overlap in ranges of scores between placentas with MVM and those without MVM. This may be due to heterogeneity of pathologic lesions present within the groups, the fact that DNA sampling was done to avoid obvious lesions, and that eoPRED was not designed to identify MVM. Nevertheless, the eoPRED score based on DNAme captures a unique MVM pathology signature.

A limitation to the use of eoPRED score is that it was developed on the Illumina EPIC v1.0 array platform and its application to newer array version remains to be shown. Nonetheless, in the development of this tool, it was shown that a model using the top 5 CpGs was as predictive as the 45 CpG tool (Fernández-Boyano et al., 2023) and we here show that all the CpGs in the tool are associated with MVM. Thus, we expect that the tool will still apply to newer array versions even if a subset of CpGs is not included on the newer arrays. We acknowledge that we only had 7 cases of EOPE in this dataset, however the high eoPRED scores in this small subset of cases support a strong association with EOPE.

In conclusion, we found that eoPRED score associates with the severity of MVM pathology, using the grading system developed by our group (Freedman et al., 2021). While some of the highest scores were seen in EOPE cases, many other placentas, particularly those associated with LOPE and PTB, also had high scores and may reflect a subset of cases with similar underlying pathology but due to other factors (maternal health, birth before the manifestation of symptoms, etc.) did not present with the clinical diagnosis of EOPE. We suggest that eoPRED score can be used to study the placenta post-delivery and it can be useful for analysing DNAme datasets that lack histopathology, preeclampsia diagnosis information, or if combining datasets with different diagnosis criteria. Importantly, the score provides a quantitative tool that can be applied to study factors associated with placental dysfunction in both preeclamptic and non-preeclamptic placentas.

## Data Availability

The dataset analyzed in this study is available on the Gene Expression Omnibus under the accession number GSE307289. Detailed histopathology information is available upon request to the authors. The function to calculate eoPRED score is available in the PlaNET package https://doi.org/doi:10.18129/B9.bioc.planet.
