# Supplementary material for: A placental DNA methylation score associated with early-onset preeclampsia and maternal vascular malperfusion pathology
Source: Front Epigenet Epigenom. 2026 Jun 5;4:1749513. doi: 10.3389/freae.2026.1749513 (PMC13441432; doi:10.3389/freae.2026.1749513)
Supplement: Supplementary file 1 [file DataSheet1.docx]

**3. Supplementary Methods**

Detailed description of MVM pathology assessment (Freedman et al. 2021)

**MVM was classified based on scores:**

•	High-grade ≥4

•	Low-grade = 2-3

•	None = 0-1

**Scores were calculated based on the number of the following pathologic features present:**

**1 point:**

•	fibrinoid necrosis/acute atherosis

•	muscularization of basal plate arterioles

•	mural hypertrophy of membrane arterioles

•	basal decidual vascular thrombus

•	single infarct

•	increased syncytial knots

•	villous agglutination

•	increased perivillous fibrin deposition

•	distal villous hypoplasia

•	retroplacental blood/hematoma

**2 points:**

•	multiple infarcts

•	retroplacental hematoma with hemosiderin or infarct

•	placental hypoplasia
