# Supplementary material for: A placental DNA methylation score associated with early-onset preeclampsia and maternal vascular malperfusion pathology
Source: Front Epigenet Epigenom. 2026 Jun 5;4:1749513. doi: 10.3389/freae.2026.1749513 (PMC13441432; doi:10.3389/freae.2026.1749513)
Supplement: Supplementary file 2 [file DataSheet2.docx]

# Supplementary Figures

**Supplementary Figure 1. Methods flowchart**

**Supplementary Figure 2. Correlation between eoPRED score and the histopathologic features of MVM and other pathology classes.**

Categorical variables coded as numeric dummy variables. Pearson’s Correlation (R) coefficients are shown for associations with p<0.05. AI = acute inflammation, CI = chronic inflammation, FVM = fetal vascular malperfusion.
