# Supplementary material for: A placental DNA methylation score associated with early-onset preeclampsia and maternal vascular malperfusion pathology
Source: Front Epigenet Epigenom. 2026 Jun 5;4:1749513. doi: 10.3389/freae.2026.1749513 (PMC13441432; doi:10.3389/freae.2026.1749513)
Supplement: Supplementary file 3 [file Table1.docx]

**Supplementary Table 1. Association between eoPRED CpGs with MVM pathology**

| **Probe ID** | **Δβ** | **p-value** | **FDR** |
| --- | --- | --- | --- |
| cg23730027 | **-0.051** | <0.001 | <0.001 |
| cg26625897 | **-0.050** | <0.001 | <0.001 |
| cg10246581 | -0.044 | <0.001 | <0.001 |
| cg16353318 | -0.043 | <0.001 | <0.001 |
| cg25103772 | -0.039 | <0.001 | <0.001 |
| cg01033642 | -0.038 | <0.001 | <0.001 |
| cg01412654 | -0.036 | <0.001 | <0.001 |
| cg05521767 | -0.030 | <0.001 | <0.001 |
| cg12779575 | -0.030 | <0.001 | <0.001 |
| cg26672098 | -0.029 | <0.001 | <0.001 |
| cg10994126 | -0.029 | <0.001 | <0.001 |
| cg12699756 | -0.029 | <0.001 | <0.001 |
| cg18236464 | -0.029 | <0.001 | <0.001 |
| cg01713086 | -0.028 | <0.001 | <0.001 |
| cg08112737 | -0.028 | <0.001 | <0.001 |
| cg22526990 | -0.027 | <0.001 | <0.001 |
| cg05663031 | -0.027 | <0.001 | <0.001 |
| cg26651514 | -0.026 | <0.001 | <0.001 |
| cg16606561 | -0.026 | <0.001 | <0.001 |
| cg07913153 | -0.026 | <0.001 | <0.001 |
| cg06501716 | -0.025 | <0.001 | <0.001 |
| cg12632411 | -0.025 | <0.001 | <0.001 |
| cg04084026 | -0.025 | <0.001 | <0.001 |
| cg26509870 | -0.025 | 0.008 | 0.009 |
| cg18584265 | -0.025 | <0.001 | <0.001 |
| cg17850498 | -0.024 | <0.001 | <0.001 |
| cg14605117 | -0.024 | <0.001 | <0.001 |
| cg02286857 | -0.023 | <0.001 | <0.001 |
| cg15282973 | -0.023 | <0.001 | 0.001 |
| cg03099780 | -0.022 | <0.001 | 0.001 |
| cg24735129 | -0.021 | <0.001 | <0.001 |
| cg23963071 | -0.021 | 0.009 | 0.010 |
| cg17066452 | -0.020 | <0.001 | <0.001 |
| cg04804772 | -0.020 | <0.001 | <0.001 |
| cg06269559 | -0.019 | <0.001 | <0.001 |
| cg17394304 | -0.019 | <0.001 | <0.001 |
| cg15799353 | -0.019 | <0.001 | <0.001 |
| cg26787199 | -0.018 | <0.001 | <0.001 |
| cg14178899 | -0.017 | 0.026 | 0.027 |
| cg15887927 | -0.016 | 0.040 | 0.040 |
| cg21545548 | -0.016 | <0.001 | 0.001 |
| cg13672136 | -0.015 | <0.001 | <0.001 |
| cg09911083 | -0.015 | <0.001 | <0.001 |
| cg24997986 | -0.011 | 0.025 | 0.027 |
| cg17035539 | -0.010 | 0.039 | 0.040 |
